# Between a Rock and a Hard Place: Balancing Physician Deception and Patient Self-Harm in the Management of Delusional Infestation

**DOI:** 10.4269/ajtmh.19-0755

**Published:** 2019-11-04

**Authors:** Matthew Grant

**Affiliations:** Department of Medicine, Section of Infectious Diseases, Yale School of Medicine, New Haven, Connecticut

In this issue of the Journal, a case report by Riggan et al. describes a patient with delusional infestation (DI, also known as delusional parasitosis) who purchased veterinary albendazole on the internet and accidentally overdosed.^1^ The patient was admitted for a syndrome consisting of alopecia, pancytopenia, and elevated transaminases. While hospitalized, he developed neutropenic fever necessitating broad-spectrum antibacterials which precipitated *Clostridioides difficile* colitis. The authors analyzed serial blood and urine specimens by liquid chromatography–mass spectrometry to document supratherapeutic concentrations of albendazole and its active metabolite.

Few publications detail toxicity following either accidental or intentional overdose of antiparasitics.^2–4^ The rarity of intentional antiparasitic overdose was suggested by a study which reviewed the Irish National Self-Harm Registry and found that only 0.3% of over 18,000 ingestions involved either antiparasitics, insecticides, or repellents.^5^ A Medline search performed for this editorial revealed only one other report of a DI patient overdosing on antiparasitics; after similarly purchasing online ivermectin from a veterinary supplier and ingesting escalating doses, that patient presented with delayed speech, intention tremor, and ataxia which resolved spontaneously over 48 hours.^6^ A 2015 case series reported unnecessary antiparasitic use by more than half of DI patients, although it was not specified what percentage of medications were prescribed.^7^ Anyone can readily purchase albendazole, ivermectin, praziquantel, nitazoxanide, pyrantel pamoate, and/or triclabendazole online without a prescription.

Whereas DI patients rarely end up hospitalized because of self-treatment, there are reports of more serious non-medication–related injuries resulting from attempts to eradicate pathogens. These include urethral stricture caused by chronic foreign body insertion requiring perineal urethrostomy, and corneal abscess due to ophthalmic application of an insecticide.^8,9^ Overall, self-treatment is so common that one descriptive study found the prevalence of self-inflicted injuries in DI patients to be 23%.^10^

Delusional infestation sufferers hold an unshakable belief that they are infected despite a workup failing to provide evidence of infection. Those with the Morgellons disease subtype feel plagued by fibers or strings emerging from their skin. Delusional infestation typically manifests with chronic and refractory dermatologic symptoms such as pruritus, burning, and/or formication. First-line treatment for DI (and for delusional disorders in general) is first- or second-generation antipsychotics. Although no randomized trials exist, a systematic review of their efficacy in primary DI showed a remission rate of 60–100%.^11^

Indirect evidence, however, suggests that few DI patients try effective pharmacotherapy. A study of DI patients presenting to the emergency room found that patients were symptomatic for a mean of more than two and half years and had seen an average of six outpatient providers before presenting to the ER.^12^ Why is there such a high incidence of refusal of proven therapy and self-harm associated with this diagnosis?

At least part of the answer can be found by looking at the provider’s experience of treating DI patients. A questionnaire-based study of European dermatologists revealed disturbing results. Three-quarters reported feeling stressed while seeing DI patients, and more than a third were subsequently accused of misdiagnosis or inadequate medical workup by a DI patient.^13^ One-tenth of these dermatologists had suffered internet trolling as a result of their DI patient interactions. There are rare examples of patients with DI who have physically assaulted and even attempted to murder their providers.^14^

How have we arrived at this place, where both the provider and the patient are predictably harmed by interacting with one another? As DI is rare (the U.S. prevalence estimated at 27 cases per 100,000 patient years)^15^ and poorly understood, medical schools and (nonpsychiatric) residency/fellowship training programs rarely include this condition in curricula. Whereas psychiatrists are well trained to manage delusional disorders and prescribe antipsychotics, DI patients are convinced that their parasitosis is real and, therefore, see no reason to seek out a mental health professional. They commonly seek relief from primary care physicians, infectious disease specialists, and dermatologists, but these providers are unable to provide them with confirmation of an infestation.

Nevertheless, patients who present with symptoms they attribute to infestation must be taken seriously. The physician must acknowledge their suffering and pursue a thorough evaluation to look for evidence of infestation or other medical condition to explain the patient’s symptomatology. However, when the diagnosis of exclusion that is DI is arrived on, the provider is confronted with an ethical dilemma.

A diagnosis that includes the word “delusional” compounds the patients’ suffering. Divulging DI’s very name to the patient destroys rapport and virtually ensures the patient will not accept antipsychotic treatment. This often leads the frustrated practitioner to invoke therapeutic privilege and withhold both the name of the diagnosis and the reason for recommending antipsychotic therapy. Without this information, the patient cannot give informed consent to treatment. This dynamic, whereas the physician must either act deceptively in hopes that the patient gets relief from antipsychotics or be honest, knowing it will likely lead to ongoing suffering, self-treatment, and at least some minor harm, is heartbreaking.

The act of naming the condition “delusional infestation” has harmed both providers and patients, as in the case described by Riggan. A first step toward mitigating the very real potential for harm would be to rename the condition in a way that does not augment the suffering of patients.
